# The Association Between Preoperative Mobility and 1-Year Survival Following Hip Fracture Surgery: A Nationwide Population Study

**DOI:** 10.3390/jcm15051764

**Published:** 2026-02-26

**Authors:** Sharon Groen, Hanne-Eva van Bremen, Jasper van Hees, Ellie B. M. Landman, Elvira R. Flikweert, Stijn A. A. N. Bolink

**Affiliations:** 1 Department of Orthopedics, Deventer Hospital, 7416 SE Deventer, The Netherlands; 2 Department of Surgery, Amsterdam Universitair Medisch Centrum, 1105 AZ Amsterdam, The Netherlands; 3 Dutch Institute for Clinical Auditing, 2333 AA Leiden, The Netherlands; 4 Department of Surgery, Deventer Hospital, 7416 SE Deventer, The Netherlands

**Keywords:** preoperative mobility, mortality, postoperative mobility

## Abstract

**Background/Objectives:** Decreased preoperative mobility increases the risk of decline and postoperative mortality in a frail patient with a hip fracture. This study investigates the correlation between preoperative mobility and 1-year mortality and between pre- and postoperative mobility. **Methods**: This retrospective, national cohort study used data from the Dutch Hip Fracture Audit (2018–2023). Excluded patients were those with an indication for a total hip arthroplasty, non-surgical treatment or missing data on mortality or preoperative mobility. Mobility was determined by the Fracture Mobility Score (FMS). A Cox proportional-hazards regression model assessed the correlation between FMS and mortality by using hazard ratios with 95% confidence intervals. A subgroup analysis was conducted for patients whose data on postoperative mobility was complete. Spearman’s test was used to assess the correlation between pre- and postoperative mobility. A *p*-value < 0.05 was considered statistically significant in both analyses. **Results**: A total of 77,185 patients were included in the study. A Cox regression model shows a stepwise increase in risk of death with a lower preoperative FMS, after correction of confounders. Those with no functional mobility showed the highest risk of death within 1 year after surgery (HR 1.86, 95% CI 1.65–2.09, *p* < 0.001). Spearman’s correlation demonstrated a moderate positive correlation between pre- and postoperative mobility, which is demonstrated by ρ = 0.49 (*p* < 0.001). **Conclusions**: Preoperative mobility seems to be an independent predictor of 1-year mortality. Additionally, this study demonstrated a moderate positive correlation between pre- and postoperative mobility.

## 1. Introduction

Posttraumatic hip fractures are a prevalent and global issue, and affect approximately 1.5 million people worldwide [1,2,3]. This number is expected to rise due to a globally increasing and aging population [1,4]. Besides the continuous optimization of hip fracture care, postoperative mortality rates remain high. In-hospital mortality rates range from 2 to 8% [5,6,7], 30-day mortality has been reported to be 4–13% [8,9,10] and 1-year mortality lies around 25–42% worldwide [9,11]. Recent evidence confirms persistently high early mortality rates following surgical treatment of hip fractures and demonstrates that mortality remains elevated in the long term, with substantial five- and ten-year mortality following hemiarthroplasty for femoral neck fractures [10].

These numbers highlight the persistent vulnerability of this patient population. Patients suffering a hip fracture frequently present with complex comorbidities and functional impairments that require a multidisciplinary approach. Additionally, shared risk factors have been identified among this population, of which reduced preoperative mobility is particularly a communal risk factor [12]. Reduced mobility is a marker of frailty, as it relies on the appropriate function of the musculoskeletal, cardiorespiratory, and nervous systems [13]. Consequently, reduced preoperative mobility may signal cognitive or physical deterioration. Patients who are classified as “non-mobile” prior to fracture have shown a higher 30-day mortality and recent evidence also indicates that pre-fracture functional status is strongly associated with adverse short-term outcomes such as readmission and postoperative morbidity [14].

To support clinical decision-making and identify patients who may derive limited benefit from surgical intervention, several prognostic models have been developed, such as the Almelo Hip Fracture Score (AHFS), the Nottingham Hip Fracture Score (NHFS) and the Zuyderland Hip Inference for Survival and Lifetime Expectancy (ZHISLE) [12,15,16]. While poor preoperative mobility is widely acknowledged as an important indicator of frailty, it is only explicitly incorporated in the AHFS. However, the AHFS only focuses on short-term outcomes (i.e., 30-day mortality) [12]. To date, no established prediction model has systematically evaluated the prognostic value of preoperative mobility on mid- or long-term mortality outcomes. This is remarkable given recent long-term mortality data after hip fracture surgery reported by Khalil et al., emphasizing the clinical relevance of predictors beyond the early postoperative period [10].

The primary aim of this study was to investigate the correlation between preoperative mobility status and mortality at 1, 6 and 12 months after traumatic hip fracture surgery. Secondly, this study investigated the correlation between preoperative and postoperative mobility.

## 2. Materials and Methods

### 2.1. Study Population

This retrospective, cohort and observational national-register study was performed using retrospective data from 66 hospitals in the Netherlands. Data was collected by the Dutch Hip Fracture Audit (DHFA) from 2018 up until and including 2023. Data was extracted from the electronic health records.

Patients included in the study were those admitted to the emergency department with a hip fracture. Patients with a pathological or periprosthetic fracture were excluded in data registration. Furthermore, we chose to exclude those patients with an indication for a total hip arthroplasty, non-surgical management, a resection arthroplasty procedure (Girdlestone), and patients whose medical records lacked information regarding the type of treatment. Only patients with a valid personal security number were included, ensuring that the mortality data is reliable. To investigate postoperative mobility, a subset of the data was used including those patients with complete postoperative mobility scores only. Baseline characteristics of this subgroup were compared with our total cohort.

### 2.2. Study Design and Data Collection

Collected variables included demographic data (age, sex, risk of malnutrition), clinical characteristics (fracture type, mobility status, comorbidities, e.g., dementia), treatment details (type of surgical procedure) and follow-up information (mortality, postoperative mobility).

Mobility status was assessed using the Fracture Mobility Score (FMS), an ordinal categorical variable (1–5) that assigns the patient into a certain category: (1) freely mobile, (2) mobile outdoors with one aid, (3) mobile outdoors with two aids or a frame, (4) some indoor mobility but never going outside without help, and (5) no functional mobility. The last category means the patient no longer has any use of the lower limbs [17]. The preoperative mobility was assessed in the emergency department throughout a questionnaire (Supplementary Materials Text S1). The postoperative mobility status was assessed 3 months (10–16 weeks) after surgery through a telephone consultation, questionnaire or visit at the outpatient clinic (Supplementary Materials Text S1).

### 2.3. Outcomes

The primary outcome was mortality up to 1 year after surgery. The secondary outcome was postoperative mobility, which was defined by the mobility status 3 months after surgery.

### 2.4. Statistical Analysis

Continuous variables were summarized as mean ± standard deviation or median with interquartile range, depending on data distribution. The distribution of data was visually assessed for normality using histogram plots. Categorical variables are presented as frequencies and percentages. Categorical variables are described using the Pearson’s Chi-square test and continuous variables using the Mann–Whitney U test.

#### 2.4.1. Correlation Between Preoperative Mobility and Postoperative Mortality

The association between the Fracture Mobility Score and all-cause mortality was assessed using a Cox proportional-hazards regression model. First, a Kaplan–Meier survival curves was constructed to visualize differences in survival probability between the preoperative mobility categories. Differences in survival between mobility groups were evaluated using the log-rank test for each interval.

Firstly, each potential covariate was analyzed through literature study. Age, gender, ASA score, risk of malnutrition, ADL dependency and dementia in the medical history have been shown to be relevant confounders for mortality [18,19]. Secondly, a univariate Cox proportional-hazards regression analysis was performed for each covariate to investigate their association with mortality. In the univariate analysis, hazard ratios (HRs) with 95% confidence intervals (CIs) were calculated to assess their individual association with mortality. Covariates with statistically significant associations were included in the multivariate analysis.

The final multivariate Cox proportional-hazards regression model was used to evaluate the association between the Fracture Mobility Score and all-cause mortality with adjustment for the previously selected covariates. HRs with 95% confidence intervals were calculated. The proportional-hazards assumption was tested using a log-minus-log plot for each mortality interval.

#### 2.4.2. Correlation Between Preoperative Mobility and Postoperative Mobility

A subset of the original dataset was formed to study postoperative mobility. A Sankey plot was created to provide a visual representation of the transitions in mobility status. To assess the levels of decline experienced per mobility category 3 months after surgery, a table was constructed that summarizes the levels of decline or improvement in Fracture Mobility Score 3 months (10–16 weeks) after surgery.

Spearman’s rank correlation coefficients were calculated to assess the strength and direction of the association between pre- and postoperative mobility scores. A *p*-value < 0.05 was regarded as statistically significant in all of the above analyses. 

All statistical analyses were performed using R (version 4.3.2).

## 3. Results

### 3.1. Cohort Description

**Figure 1:**
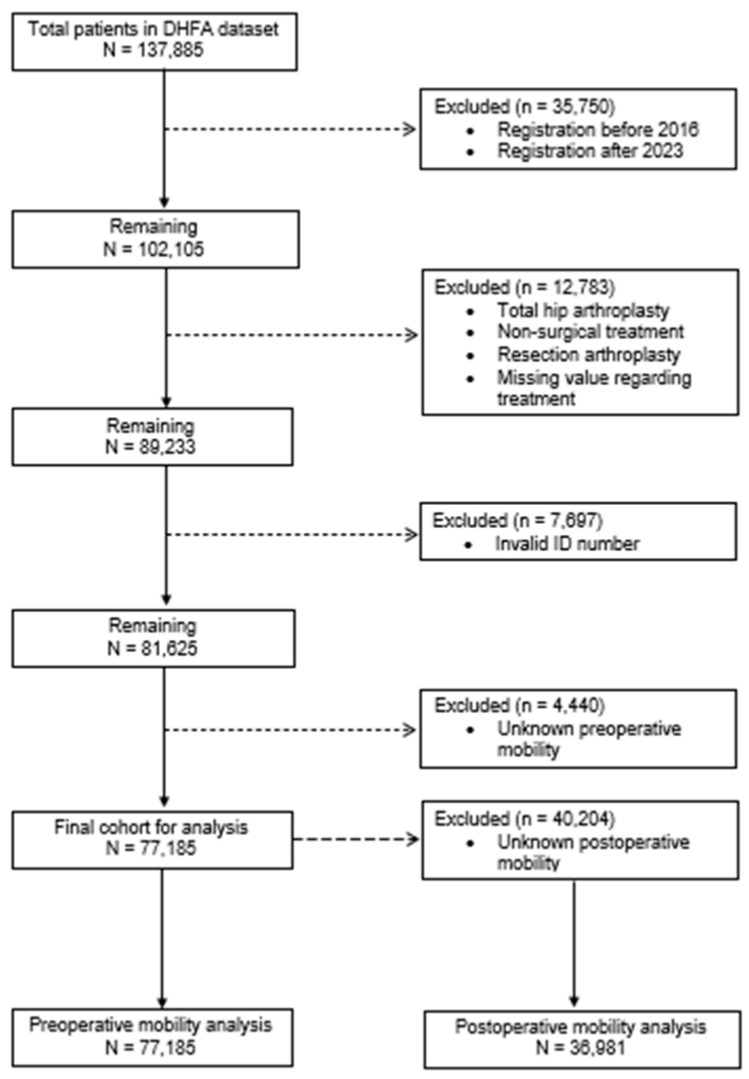
Flowchart of patient inclusion and exclusion. Abbreviations: DHFA = Dutch Hip Fracture Audit, *n* = number of patients.

A total of 137,885 patients were registered in the DHFA database. After applying exclusion criteria, 77,185 patients were included in the main analysis. A total of 40,204 patients (52.1%) had missing postoperative mobility data. Therefore, the subgroup analysis regarding postoperative mobility consisted of 36,981 patients (47.9% of the total cohort). The numbers outlining patient inclusion and exclusion are presented in a flowchart (Figure 1).

The total cohort (77,185 patients) demonstrated a median age of 82 years (IQR 73–88, *p* < 0.001) and 65.1% of patients were female. In this cohort, 40.7% of patients were diagnosed with a trochanteric femur fracture, 17.7% an undisplaced femoral neck fracture, 35.7% a displaced femoral neck fracture, and 4.4% a subtrochanteric femur fracture. Regarding surgical management, a cephalomedullary nail was performed in 41.0% of cases, a hemiarthroplasty in 39.0% of cases, a sliding hip screw in 14.5% of cases and a cannulated screw fixation in 5.3% of cases (Table 1).

Prior to surgery, the largest proportion of patients (49.7%) was freely mobile (FMS 1), whereas those patients whose preoperative mobility was described as mobile outdoors with one aid (FMS 2) made up 7.6% of the total population. Patients who were mobile outdoors but with the help of two aids or a frame (FMS 3) represented 33.0% of the population. Those who were only mobile indoors (FMS 4) accounted for 8.1% of the total population. The group of patients who had no longer any use of the lower limbs, described as no functional mobility (FMS 5), consisted of 1295 patients (1.7%) (Table 1).

Regarding demographic differences, the freely mobile group (FMS 1) had a significant lower median age of 77 years (IQR 68–83), in comparison to the other mobility groups. Furthermore, in the freely mobile group only 7.0% had a reported dementia diagnosis. When comparing these characteristics to the group of patients who were mobile outdoors with two aids (FMS 3), 40.2% had a reported dementia diagnosis (*p* < 0.001) (Table 1).

Furthermore, a difference was found between the mobility groups with regard to ADL dependency. With decreasing preoperative mobility, ADL dependency was more frequent. Meanwhile, in the freely mobile group (FMS 1), 17.1% of patients were ADL-dependent; in the some-indoor-mobility group (FMS 4) ADL dependency was 80.1% (*p* < 0.001). Furthermore, decreased preoperative mobility was associated with higher ASA scores. In the freely mobile group, 44.1% of patients had an ASA score of III, IV or V, which was significantly lower than the 83.2% in the some-indoor-mobility group (FMS 4) and the 73.3% in the no-functional-mobility group (FMS 5) (Table 1).

Baseline characteristics of included patients regarding the subgroup analysis (*n* = 39,981, 47.9%) were compared with excluded patients; no relevant differences in baseline characteristics were found.

### 3.2. Fracture Mobility Score in Correlation to Mortality

**Figure 2:**
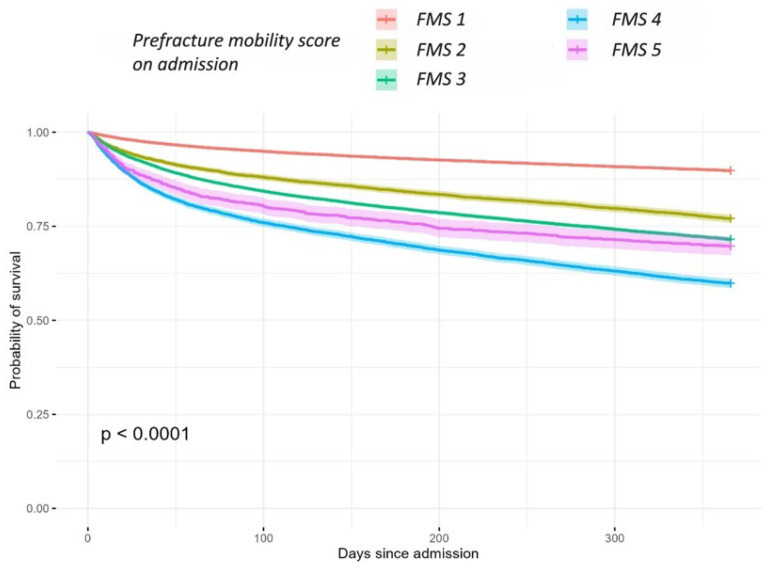
Kaplan–Meier curve illustrating 1-year survival probability between different mobility groups.

Visual inspection of the Kaplan–Meier curves reveals a difference, indicating that the freely mobile group (FMS 1) had the highest probability of survival up to one year. The group of patients with some indoor mobility (FMS 4) had the lowest probability of survival, as shown in Figure 2 (log-rank test: 30 days X^2^ = 1839; 6 months X^2^ = 3945; 1 year X^2^ = 5409, *p* < 0.001) (Figure 2).

All potential covariates found in the literature (age, gender, ASA score, dementia diagnosis, risk of malnutrition and ADL dependency) were individually analyzed by univariate analysis (Supplementary Materials Table S1). All covariates demonstrated statistical significance (*p* < 0.001) and were therefore included in the multivariate analysis.

Compared to individuals who were freely mobile (FMS 1), those who were mobile outdoors with one walking aid (FMS 2) had a hazard ratio (HR) of 1.21 regarding mortality at 30 days and 6 months (95% CI 1.10–1.32, *p* < 0.001) and a HR of 1.26 (95% CI 1.17–1.36, *p* < 0.001) for mortality after 1 year. Similarly, individuals who were mobile outdoors with two aids (FMS 3) had a HR of 1.40 at 30 days and 6 months (95% CI 1.33–1.49, *p* < 0.001) and a HR of 1.43 (95% CI 1.37–1.51, *p* < 0.001) at one year. Individuals who were mobile indoors but could never go outside without help (FMS 4) had a HR of 1.79 at 30 days and 6 months (95% CI 1.66–1.92, *p* < 0.001) and a HR of 1.81 (95% CI 1.70–1.93, *p* < 0.001) after one year (Table 2).

Patients who had no functional mobility (FMS 5) had the highest risk for 30-day, 6-month and 1-year mortality after adjustment for confounders (age, gender, ASA score, dementia, risk of malnutrition and ADL dependency). For this group, the risk of death within 30 days and 6 months was nearly double compared to the reference group (HR 1.98; 95% CI 1.74–2.26, *p* < 0.001) for 30 days and 6 months. The risk remained elevated at the interval of one-year mortality, whereas the hazard ratio was still 1.86 (95% CI 1.62–2.09, *p* < 0.001) (Table 2).

### 3.3. Correlation Between Preoperative and Postoperative Mobility

**Figure 3:**
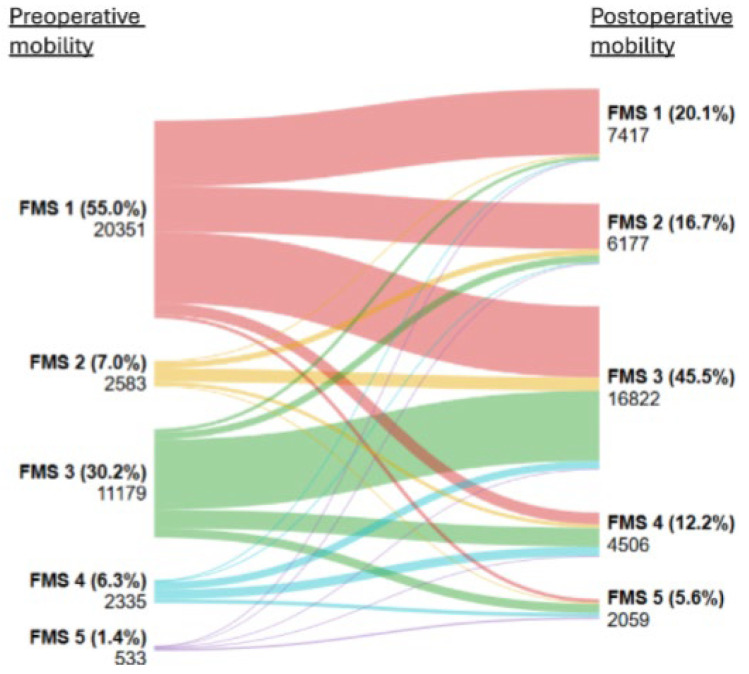
Sankey plot illustrating transitions from pre- to postoperative mobility. Left are all preoperative mobility groups with each a different color. Right are all postoperative mobility groups. All given numbers (%) are a portion of the total population (*n* = 36,981). Abbreviations: FMS = Fracture Mobility Score.

The subset for analysis, regarding the correlation between pre- and postoperative mobility, consisted of 36.981 patients (47.9% of the total cohort). Based on preoperative mobility status, 20,351 patients (55.0%) were classified as freely mobile (FMS 1), 2583 (7.0%) as mobile outdoors with one aid (FMS 2), 11,179 (30.2%) as mobile outdoors with two aids (FMS 3), 2335 (6.3%) as having some indoor mobility (FMS 4), and 533 (11.4%) as having no functional mobility (FMS 5). Transitions from pre- to postoperative mobility are visualized in a Sankey plot (Figure 3).

When analyzing postoperative decline in Fracture Mobility Score at 3 months among patients who were freely mobile before surgery (FMS 1), 6774 patients (33.3%) showed no decline, while one-level decline occurred in 4680 patients (23.0%). Most common was a two-level decline, affecting 7324 patients (36.0%). A three-level decline was observed in 1197 (5.9%) and four-level decline in 376 (1.8%) (Table 3). In total, more than 60% of patients experienced a decline in mobility after 3 months following surgery.

For those patients who preoperatively needed one aid while outdoors (FMS 2), no decline in FMS after 3 months was observed in 629 patients (24.4%). A decline of one level was seen in 1354 patients (52.5%), while a two-level decline was observed in 362 patients (14.0%). Less frequent was a three-level decline, demonstrated in 106 patients (4.1%) (Table 3).

For the preoperative mobility category of patients moving outdoors with the help of two aids (FMS 3), 7228 patients (64.7%) maintained their preoperative level after 3 months. A one-level decline was observed in 1932 patients (17.3%) and only 940 patients (8.4%) demonstrated a two-level decline (Table 3).

Moreover, the group that showed only some indoor mobility before surgery (FMS 4) had a total of 963 patients (41.3%) that showed no decline in FMS. One-level decline for this group was seen in 410 patients (17.6%). For patients who already no longer had any functional mobility at the time of admission to the emergency department (FMS 5), no further decline in FMS was possible, as this already represents the lowest achievable mobility level (Table 3).

In addition to reduced mobility, a substantial loss of independence was seen. Prior to fracture, most patients were independently moving outdoors, corresponding to Functional Mobility Scale (FMS) levels 1–3. Preoperatively, 92.2% of the patients were able to walk outdoors independently, meaning without assistance from caregivers. At three months post operation, this number had declined to 82.3% of all patients. Consequently, approximately 10% of the total population transitioned from independent (FMS 1–3) to dependent (FMS 4–5) outdoor mobility, requiring assistance from family members or caregivers.

In addition to postoperative decline, improvement in FMS was observed in a subset of patients. In the preoperative group that was classified as mobile outdoors with one aid (FMS 2), 132 patients (5.1%) improved postoperatively to freely mobile (FMS 1). Among patients who were mobile outdoors with two aids preoperatively (FMS 3), a one-level improvement was found in 730 patients (6.5%) and a two-level improvement was found in 349 patients (3.1%). In the preoperative group of some indoor mobility (FMS 4), a one-level improvement was found in 784 patients (33.6%), a two-level improvement was found in 112 patients (4.8%) and a three-level improvement was found in 66 patients (2.8%). Finally, among those who showed no functional mobility before surgery (FMS 5), postoperative improvements were observed in 2479 patients (6.7%). An improvement of one level in FMS was observed in 52 patients (9.8%) who improved to some indoor mobility (FMS 4); a two-level improvement was found in 132 patients (24.7%) who became mobile outdoors with two aids (FMS 3); a three-level improvement was found in 26 patients (4.9%) who became mobile outdoors with one aid (FMS 2); and a four-level improvement was found for 96 patients (18.0%) who became freely mobile after surgery.

Spearman’s rank-order correlation demonstrated a moderately positive correlation between pre- and postoperative mobility, which is demonstrated by ρ = 0.49 (*p* < 0.001) (Supplementary Materials Table S2).

## 4. Discussion

### 4.1. Key Findings

In this nationwide cohort study, a worse preoperative mobility status was associated with a progressively higher risk of mortality at 30 days, 6 months and 1 year. These findings confirm that vulnerability extends beyond the acute phase and persists well into the longer postoperative period.

Current mortality prediction models acknowledge the importance of frailty-related factors; however, they largely focus on early mortality and some omit preoperative mobility as an important predictor [12,15,16]. Given the observed association between preoperative mobility and mid- to long-term mortality in this study, incorporating mobility into prognostic frameworks may improve risk stratification and support more individualized management strategies.

In addition to mortality, previous research has shown that patients with poor pre-fracture functional level tend to have a worse early postoperative functional recovery [17]. In a frail population, even a minor deterioration can be associated with a loss of independence, which may be exacerbated by surgical stress or postoperative complications [20].

Only a limited number of small-scale studies have examined the relation between pre- and postoperative mobility [21,22]. The moderate correlation between preoperative and postoperative mobility found in this study indicates that many patients fail to regain their baseline level of independence at least three months postoperatively, which is consistent with previous findings [21,23] and aligns with the results of our study. As observed in this study, more than 60% of patients experienced a decline in mobility after 3 months following surgery. In addition to reduced mobility, a substantial loss of independence was seen. Specifically, the proportion of patients able to move outdoors without assistance from caregivers (FMS 1–3) decreased from 92.3% preoperatively to 82.3% within three months after surgery.

These findings are highly relevant in the context of contemporary hip fracture management, which increasingly emphasizes multidisciplinary care pathways. Older hip fracture patients often present with complex comorbidities and limited physiological reserves, requiring coordinated involvement of orthopedic surgeons, geriatricians, anesthesiologists, and rehabilitation specialists. Within such models of care, mobility emerges not only as a postoperative outcome but also as a key prognostic marker that can inform perioperative decision-making, postoperative monitoring, and rehabilitation planning.

### 4.2. Strengths and Limitations

The strengths of this study include its large nationwide cohort, standardized data collection across multiple centers, and robust multivariable analyses. However, several limitations should be acknowledged. Mobility was assessed using self-reported measures, which may be subject to misclassification. Although no relevant differences in baseline characteristics were found, exclusion of patients with missing postoperative mobility in our subgroup analysis may have introduced selection and survivorship bias. Due to exclusion of patients with an indication for a total hip arthroplasty, inclusion bias may be present. However, as these patients had the highest mobility level (FMS 1), this is unlikely to have affected the results.

Furthermore, postoperative mobility assessments did not extend beyond 3 months whereas full functional recovery is unlikely to have been achieved by then. In addition, improvement of mobility within 3 months after surgery remains poorly understood. Residual confounding cannot be fully excluded, and complete-case analysis reduced the final analytic sample.

### 4.3. Implications for Future Research

This study, along with previous research, primarily focused on identifying preoperative factors, also called predictors, that influence early mortality. The underlying reasons for later mortality, after 30 days but within 1 year, remain poorly understood. A key question is, what are distinguishing factors for those who die outside the hospital within the first year in comparison to those who survive? We propose that identifying risk factors, such as postoperative mobility, should be assessed during the acute postoperative period, which begins upon admission and lasts approximately two weeks. This period may represent a critical window in which healthcare professionals can make a difference for a specific patient group: those who die after 30 days and within the first postoperative year.

Future research should therefore shift towards exploring modifiable postoperative factors such as postoperative mobility and sustained functional decline. Additionally, objective measurement tools, such as wearable motion sensors, could provide a more detailed understanding of postoperative mobility at home.

## 5. Conclusions

To conclude, this study demonstrates the relationship between preoperative mobility and postoperative mortality in patients undergoing surgery for a traumatic hip fracture. Preoperative mobility is an independent predictor of 30-day, 6-month and 1-year mortality, whereas patients with reduced mobility prior to surgery demonstrate a progressively higher risk of death compared to those who were fully mobile. The data show a stepwise increase in the risk of death as preoperative mobility decreases. Additionally, this study shows a moderate positive correlation between preoperative mobility and mobility at 3 months after traumatic hip surgery, indicating that other factors than a patient’s preoperative mobility status may influence individual postoperative recovery.

## Figures and Tables

**Table 1:** Patient characteristics and types of treatment.

Characteristic	Total Population	FMS 1 Freely Mobile	FMS 2 Mobile Outdoors with One Aid	FMS 3 Mobile Outdoors with Two Aids	FMS 4 Some Indoor Mobility	FMS 5 No Functional Mobility	*p*-Value
Total, *n* (%)	77,185 (100)	38,366 (49.7)	5854 (7.6)	25,442 (33.0)	6228 (8.1)	1295 (1.7)	
**Age**, years ^a^	82 [73, 88]	77 [68, 83]	85 [78, 89]	86 [81, 90]	85 [79, 90]	80 [71, 87]	<0.001
**Sex**, *n* (%)							<0.001
Male	26,790 (34.7)	15,402 (40.1)	2254 (38.5)	6745 (26.5)	1873 (30.1)	516 (39.8)	
Female	50,269 (65.1)	22,893 (59.7)	3593 (61.4)	18,657 (73.3)	4349 (69.8)	777 (60.0)	
NA	126 (0.2)	71 (0.2)	7 (0.1)	40 (0.2)	6 (0.1)	2 (0.2)	
**Risk of malnutrition**, *n* (%)							<0.001
No risk	62,603 (81.1)	32,689 (85.2)	4694 (80.2)	19,677 (77.3)	4527 (72.7)	1016 (78.5)	
Mild risk	2972 (3.9)	1088 (2.8)	214 (3.7)	1228 (4.8)	379 (6.1)	63 (4.9)	
High risk	7335 (9.5)	2477 (6.5)	630 (10.8)	3144 (12.4)	933 (15.0)	151 (11.7)	
NA	4275 (5.5)	2112 (5.5)	316 (5.4)	1393 (5.5)	389 (6.2)	65 (5.0)	
**Dementia**, *n* (%)	11,720 (15.2)	2683 (7.0)	798 (13.6)	5416 (21.3)	2504 (40.2)	319 (24.6)	<0.001
**ADL dependency**, *n* (%)							<0.001
Independent	42,781 (55.4)	30,166 (78.6)	2850 (48.7)	8415 (33.1)	968 (15.5)	382 (29.5)	
Dependent	30,945 (40.1)	6568 (17.1)	2718 (46.4)	15,820 (62.2)	4987 (80.1)	852 (65.8)	
NA	3459 (4.5)	1632 (4.3)	286 (4.9)	1207 (4.7)	273 (4.4)	61 (4.7)	
**ASA score**, *n* (%)							<0.001
I & II	29,624 (38.4)	21,048 (54.9)	1599 (27.3)	5675 (22.3)	980 (15.7)	322 (24.9)	
III, IV & V	46,678 (60.5)	16,917 (44.1)	4155 (71.0)	19,473 (76.5)	5184 (83.2)	949 (73.3)	
NA	883 (1.1)	401 (1.0)	100 (1.7)	294 (1.2)	64 (1.0)	24 (1.9)	
**Type of fracture**, *n* (%)							<0.001
Trochanteric femur fracture	31,368 (40.7)	14,534 (37.9)	2355 (40.3)	11,231 (44.1)	2714 (43.6)	534 (41.2)	
Displaced femoral neck fracture	27,556 (35.7)	13,182 (34.4)	2109 (36.0)	9456 (37.2)	2390 (38.4)	419 (32.4)	
Undisplaced femoral neck fracture	13,631 (17.7)	8317 (21.7)	988 (16.9)	3349 (13.2)	756 (12.1)	221 (17.1)	
Subtrochanteric femur fracture	3362 (4.4)	1732 (4.5)	224 (3.8)	1057 (4.2)	269 (4.3)	80 (6.2)	
Unspecified	884 (1.1)	432 (1.1)	144 (2.5)	206 (0.8)	66 (1.1)	36 (2.8)	
NA	384 (0.5)	169(0.4)	34 (0.6)	143 (0.6)	33 (0.5)	5 (0.4)	
**Type of operative treatment**							<0.001
Cephalomedullary nail	31,758 (41.1)	14,727 (38.4)	2353 (40.2)	11,285 (44.4)	2808 (45.1)	585 (45.2)	
Hemiarthroplasty	30,136 (39.0)	12,728 (33.2)	2696 (46.1)	11,472 (45.1)	2757 (44.3)	483 (37.3)	
Cannulated screws	4096 (5.3)	2969 (7.7)	196 (3.3)	711 (2.8)	177 (2.8)	43 (3.3)	
Sliding hip screw	11,195 (14.5)	7942 (20.7)	609 (10.4)	1974 (7.8)	486 (7.8)	184 (14.2)	

^a.^ Median with IQR (interquartile range). Risk of malnutrition was assessed using the Short Nutritional Assessment Questionnaire (SNAQ). ADL dependency was assessed using KATZ-ADL (Katz Index of Independence in Activities of Daily Living). Abbreviations: FMS = Fracture Mobility Score, *n* = number of patients, ADL = Activities of Daily Living, ASA = American Society of Anesthesiologists.

**Table 2:** Multivariate analysis for all mortality intervals.

Variable	HR (95% CI) for 30-Days Mortality	HR (95% CI) for 6-Month Mortality	HR (95% CI) for 1-Year Mortality	*p*-Value
Preoperative Mobility				
FMS 2	1.21 (1.10–1.32)	1.21 (1.10–1.32)	1.26 (1.17–1.36)	<0.001
FMS 3	1.40 (1.33–1.49)	1.40 (1.33–1.49)	1.43 (1.37–1.51)	<0.001
FMS 4	1.79 (1.66–1.92)	1.79 (1.66–1.92)	1.81 (1.70–1.93)	<0.001
FMS 5	1.98 (1.74–2.26)	1.98 (1.74–2.26)	1.86 (1.65–2.09)	<0.001

Hazard ratios were calculated using multivariate Cox regression with the freely mobile group (FMS 1) as reference group. Adjustments were made for age, gender, ASA score, dementia diagnosis, risk of malnutrition and ADL dependency. Abbreviations: HR = hazard ratio, CI = confidence interval, FMS = Fracture Mobility Score.

**Table 3:** Levels of decline or improvement in FMS 3 months after surgery.

Preoperative Mobility						
	Total Population (*n* = 36,981)	FMS 1 (*n* = 20,351)	FMS 2 (*n* = 2583)	FMS 3 (*n* = 11,179)	FMS 4 (*n* = 2335)	FMS 5 (*n* = 533)
Decline in FMS after 3 months						
Zero-level decline	15,821 (42.8%)	6774 (33.3%)	629 (24.4%)	7228 (64.7%)	963 (41.3%)	227 (42.6%)
One-level decline	8376 (22.6%)	4680 (23.0%)	1354 (52.5%)	1932 (17.3%)	410 (17.6)	
Two-level decline	8626 (23.3%)	7324 (36.0%)	362 (14.0%)	940 (8.4%)		
Three-level decline	1301 (3.5%)	1197 (5.9%)	106 (4.1%)			
Four-level decline	376 (1%)	376 (1.8%)				
Improvement	2479 (6.7%)		132 (5.1%)	1079 (9.6%)	962 (41.2%)	306 (57.4%)

Gray indicates no further decline or improvement possible.

## Data Availability

By Dutch law, ethical approval was not required for this study as data is fully anonymized. The medical ethical committee approved research using DHFA data, and this research was not deemed subject to the Medical Research Involving Human Subjects Act in compliance with Dutch regulations.

## Supplementary Materials

The following supporting information can be downloaded at: https://www.mdpi.com/article/10.3390/jcm15051764/s1, Text S1: Fracture Mobility Score questionnaire for patients. Table S1: Univariate analysis results for 30-day, 6-month and 1-year mortality. Table S2: Spearman’s rank correlation test.
