# Supplementary material for: The Association Between Preoperative Mobility and 1-Year Survival Following Hip Fracture Surgery: A Nationwide Population Study
Source: J Clin Med. 2026 Feb 26;15(5):1764. doi: 10.3390/jcm15051764 (PMC12986506; doi:10.3390/jcm15051764)
Supplement: Supplementary file 1 [file jcm-15-01764-s001.zip › jcm-4134014-supplementary.pdf]

Journal of
Clinical Medicine

MDPI

Article

The Association Between Preoperative Mobility and 1-Year Survival Following Hip Fracture Surgery: A Nationwide Population Study

Sharon Groen [1]*, Hanne-Eva van Bremen [2,3] , Jasper van Hees [1], Ellie B.M. Landman [1] , Elvira R. Flikweert [3,4] and Stijn A.A.N. Bolink [1] on behalf of the Dutch Hip Fracture Audit Group

1 Department of Orthopedics, Deventer hospital, 7416 SE, Deventer, The Netherlands
2 Department of Surgery, Amsterdam Universitair Medisch Centrum, 1105 AZ, Amsterdam, The Netherlands
3 Dutch Institute for Clinical Auditing, 2333 AA, Leiden, The Netherlands
4 Department of Surgery, Deventer hospital, 7416 SE, Deventer, The Netherlands
* Correspondence: sharon.groen99@gmail.com; Tel.: 0031619495278

Supplementary files

Text S1 Fracture Mobility Score questionnaire for patients

Instructions: please answer the question below based on your current level of mobility. Tick one box that describes your situation.

☐ I am able to walk outdoors independently without using any walking aids.

☐ I am able to walk outdoors independently, but I need one walking aid (such as a cane and walker).

☐ I am able to walk outdoors independently, but I need two walking aids (such as a cane and walker) or a frame.

☐ I am able to mobilize indoors independently, but I can never go outside without help from a caregiver or a family member.

☐ I no longer have any ability to walk since I am mostly chair- or bed-bound.

Text S2 univariate analysis results

Table S1 Univariate analysis results for 30-day, 6-month and 1-year mortality

Variable	*HR (95% CI) for 30-days mortality*	*HR (95% CI) for 6-month mortality*	*HR (95% CI) for 1-year mortality*	P-value
Age	1.07 (1.07-1.08)	1.07 (1.07-1.07)	1.07 (1.06-1.07)	< 0.001
Female sex	0.66 (0.62-0.71)	0.74 (0.70-0.77)	0.76 (0.74-0.79)	< 0.001
ASA-score III, IV or V	5.20 (4.73-5.73)	4.29 (4.06-4.53)	3.92 (3.75-4.10)	< 0.001
High risk of malnutrition	2.31 (2.14-2.50)	2.37 (2.26-2.50)	2.29 (2.19-2.38)	< 0.001
Dementia	3.66 (3.43-3.90)	3.29 (3.16-3.43)	3.18 (3.06-3.29)	< 0.001
ADL-dependency	4.10 (3.82-4.40)	3.70 (3.55-3.86)	3.53 (3.42-3.66)	< 0.001

Hazard ratio's were calculated using univariate Cox regression with the following groups as reference groups: male sex, ASA-score I or II, no risk of malnutrition, no dementia diagnosis and ADL-independency. Abbreviations: HR = Hazard Ratio, CI = Confidence interval, ASA = American Society of Anesthesiologists, ADL = Activities of Daily Living.

Table S2. Spearman's rank correlation test.

			**Postoperative mobility**
Spearman's rank	**Preoperative mobility**	Correlation coefficient (ϱ)	0.493
		P-value	<0.001
		Hypothesis	ϱ ≠ 0
